# Correlation of Online Physician Rating Subscores and Association With Overall Satisfaction: Observational Study of 212,933 Providers

**DOI:** 10.2196/11258

**Published:** 2020-10-27

**Authors:** Hanson Hanqing Zhao, Michael Luu, Brennan Spiegel, Timothy John Daskivich

**Affiliations:** 1 Division of Urology Cedars-Sinai Medical Center Los Angeles, CA United States; 2 Center for Outcomes Research and Education Cedars-Sinai Medical Center Los Angeles, CA United States; 3 Division of Health Services Research Department of Medicine Cedars-Sinai Medical Center Los Angeles, CA United States; 4 Department of Health Policy and Management University of California, Los Angeles Los Angeles, CA United States

**Keywords:** online ratings, Healthgrades, physician ratings

## Abstract

**Background:**

Online physician rating websites commonly ask consumers to rate providers across multiple physician-based (eg, spending sufficient time, listening) and office-based (eg, appointment scheduling, friendliness) subdimensions of care in addition to overall satisfaction. However, it is unclear if consumers can differentiate between the various rated subdimensions of physicians. It is also unclear how each subdimension is related to overall satisfaction.

**Objective:**

The objectives of our study were to determine the correlation of physician-based and office-based subdimensions of care and the association of each with overall satisfaction.

**Methods:**

We sampled 212,933 providers from the Healthgrades website and calculated average provider metrics for overall satisfaction (likelihood to recommend doctor), physician-based subdimensions (trust in physician, ability to explain, ability to listen and answer questions, and spending adequate time), and office-based subdimensions (ease of scheduling, office environment, staff friendliness, and wait time). We used Spearman rank correlation to assess correlation between subdimension ratings. Factor analysis was used to identify potential latent factors predicting overall satisfaction. Univariate and multivariable linear regression were performed to assess the effect of physician and office-based factors on overall satisfaction.

**Results:**

Physician-based metrics were highly correlated with each other (*r*=.95 to .98, *P*<.001), as were office-based metrics (*r*=.84 to .88, *P*<.001). Correlations between physician-based and office-based ratings were less robust (*r*=.79 to .81, *P*<.001). Factor analysis identified two factors, clearly distinguishing between physician-based metrics (factor loading = 0.84 to 0.88) and office-based metrics (factor loading = 0.76 to 0.84). In multivariable linear regression analysis, the composite factor representing physician-based metrics (0.65, 95% CI 0.65 to 0.65) was more strongly associated with overall satisfaction than the factor representing office-based metrics (0.42, 95% CI 0.42 to 0.42). These factors eclipsed other demographic variables in predicting overall satisfaction.

**Conclusions:**

Consumers do not differentiate between commonly assessed subdimensions of physician-based care or subdimensions of office-based care, but composite factors representing these broader categories are associated with overall satisfaction. These findings argue for a simpler ratings system based on two metrics: one addressing physician-based aspects of care and another addressing office-based aspects of care.

## Introduction

Online physician ratings websites have become an increasingly influential source of information for health care consumers [1-3]. While rating websites provide transparency and a platform for patient feedback, there is little data supporting their validity and utility in identifying high-quality care [4-6]. Nevertheless, studies suggest that consumers believe these sites are important in choosing a physician [7]. A survey of 1000 surgical patients at Mayo Clinic found that 75% would choose a physician and 88% would avoid a physician based on ratings alone [8]. As testament to the growing acceptance and trust in consumer ratings, payers and institutions now list commercial consumer ratings as part of online provider listings, and a percentage of Medicare payments are redistributed to hospitals with higher quality metrics and better patient evaluations [9-11]. Of the top 20 hospitals in a recent US News & World Report ranking, 10 of them currently display ratings for their providers [12].

Most ratings websites prominently feature overall satisfaction scores on a 5-star Likert scale [13,14]. To improve clarity, some websites additionally ask consumers to rate physicians on specific subdimensions of care related to the physician’s bedside manner (eg, level of trust in provider’s decisions, how well the provider explains medical conditions, how well the provider listens and answers questions, spending sufficient time with patients) and office (eg, ease of scheduling, staff friendliness, total wait time, office environment) [15-17]. Although there is good face validity in asking consumers to rate physicians on these discrete, service-related aspects of care, there is a lack of data showing that patients actually distinguish between these subdimensions. Furthermore, the individual contribution of each subdimension to overall satisfaction is unknown.

In this study, we analyzed a large, heterogeneous sample of quantitative online reviews to determine if consumers are able to parse different components of the patient experience and identify physician and office characteristics that predict higher overall satisfaction scores. We hypothesized that all physician-related scores would be highly correlated, since patients are asked to rate subdimensions that are all related to bedside manner, while office-based scores may vary, as they measure distinct aspects of care that are unrelated.

## Methods

### Data Source

We sampled online consumer reviews for providers in the United States from the Healthgrades website using a method that has previously been described [18]. The dataset consisted of 2.7 million reviews for 830,308 providers up to March 31, 2017. These data were linked with demographic information from the US Centers for Medicare & Medicaid Services Physician Compare website using National Provider Identifier numbers: this information included medical specialty, region, gender, and year of graduation from medical school. In order to sample physicians with an adequate number of reviews, we excluded physicians with 4 or fewer reviews (n=611,013). We also excluded physicians who were missing information on their primary specialty (n=1813) or who were identified as nursing or nonclinical specialty providers (n=4549). Our final analytic sample comprised 212,933 physicians. The study was approved by the Cedars-Sinai IRB.

### Physician Rating Selection

Average provider metrics across all reviews on a 5-star Likert scale were collected for overall satisfaction and subdimensions of perceived physician quality including level of trust in provider’s decisions, how well the provider explains medical conditions, how well the provider listens and answers questions, and spending the appropriate amount of time with patients (see Multimedia Appendix 1 for a screenshot of the homepage and a sample review). Office-based metrics were also collected across subdimensions of ease of scheduling urgent appointments, office environment, staff friendliness and courteousness, and total wait time.

### Statistical Analysis

Physician demographics were described using median and interquartile range for continuous variables and counts with percentages for categorical variables.

Spearman rank correlation coefficient was used to assess the correlation between overall satisfaction, physician-based ratings subdimensions, and office-based ratings subdimensions. Additionally, a scatter plot matrix was used to visually depict the strength of association between pairings of overall satisfaction, physician-based, and office-based metrics.

To identify potential latent factors among our physician-based and office-based metrics, exploratory and confirmatory factor analysis was conducted [19]. Sampling adequacy was confirmed by the Kaiser-Meyer-Olkin statistic (0.93) to examine the appropriateness of the sample size for conducting exploratory factor analysis [20]. Sampling adequacy values between .80 to .90 are considered excellent, where values between .50 and .60 are considered marginal, and below .50 considered unacceptable [21]. The Bartlett test of sphericity was also conducted to test the null hypothesis of an identify matrix and the suitability for factor extraction (*P*<.001) [22].

**Figure 1:**
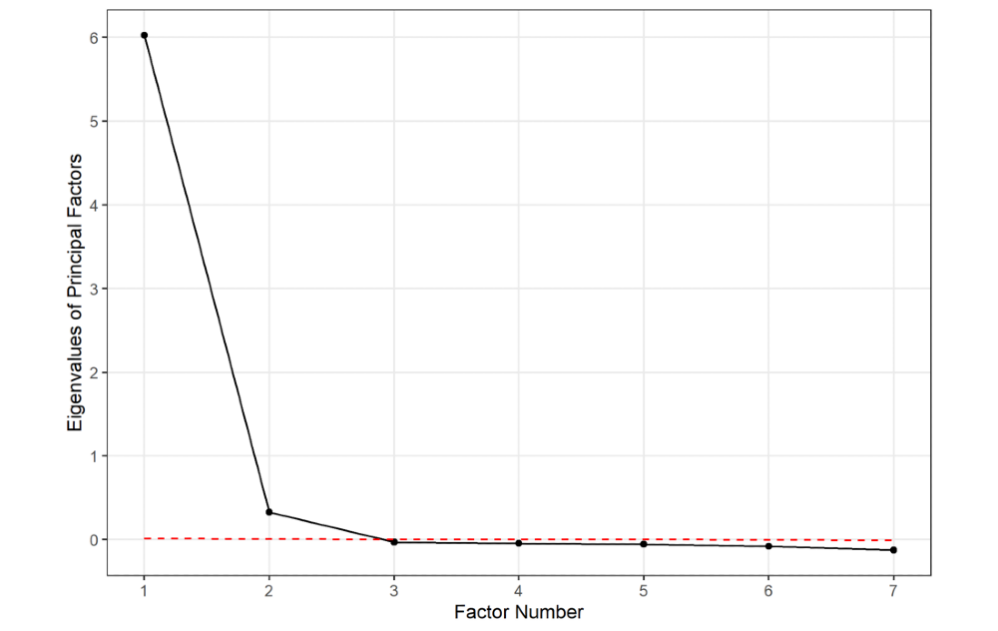
Scree plot.

To determine the number of potential latent factors in our sample, we conducted the Horn parallel analysis [23]. Parallel analysis involves the generation of a random dataset with the same number of observations and variables. The eigenvalues and correlation matrix are computed from this dataset and compared with results of the eigenvalues from factor extraction. The point at which the eigenvalues from the random data exceed the values from factor extraction indicates that any further factors encompass primarily random noise. We identified the point at which the decrease in eigenvalues became negligible on the scree plot (Figure 1), which revealed a 2-factor solution. Factor extraction was conducted based on the orthogonal varimax rotation and extracted using the maximum likelihood. Exploratory factor analysis was performed using a 1-, 2-, and 3-factor solution with the resulting cumulative variance explained of .840, .929, and .932, respectively.

Confirmatory factor analysis was further conducted to test 1-, 2-, and 3-factor models of overall patient satisfaction. The hypothesized latent factor structure is overall physician satisfaction measured by physician ratings subdomains. The confirmatory factor analysis model was fit using lavaan version 0.5-23 (Rosseel, 2012) and showed acceptable goodness of fit (Tucker-Lewis index 0.989, comparative fit index .993, root mean square error of approximation 0.087 [90% CI 0.086 to 0.088], goodness of fit index 0.975, adjusted goodness of fit index 0.945, and standardized root mean square residual 0.01) with all subdomains loading significantly on their hypothesized latent factors (*P*<.001). Furthermore, discriminatory validity of the measures has been assessed using composite reliability and the average variance extracted for the 2-factor solution. Composite reliability among the physician satisfaction measures (0.99) and office staff satisfaction measures (0.95) both showed high internal consistency [24]. The average variance extracted also showed a high amount of variance captured by the two factors solution for both physician satisfaction (0.97) and office staff satisfaction (0.87) [25]. In contrast, the fit of the 1-factor model provided a lower chi-square (262,257 vs 20,950), lower Akaike information criterion (219,065 vs 460,370), lower root mean square error of approximation (0.087 vs 0.297), and higher comparative fit index (0.993 vs 0.912) and goodness of fit index (0.975 vs 0.730) in comparison with the 2-factor model.

To assess the relative impact of physician-based and office-based metrics on overall satisfaction, univariate linear regression was performed [26]. Additionally, a multivariable linear regression model was performed regressing the saved factor scores extracted from exploratory factor analysis on overall satisfaction adjusting for all physician demographics.

All statistical analyses were performed using R version 3.5.1 (R Foundation for Statistical Computing) with 2-sided test and significance level of .05 [27].

## Results

Physician characteristics are summarized in Table 1. The majority of our sample were medical specialists (128,678/212,933, 60.43%) from the southern United States (80,751/212,701, 37.96%) who graduated from medical school after 1985 (146,246/209,095, 69.94%). Median scores for overall satisfaction, physician-based metrics, and office-based metrics were universally high (range 4.1 to 4.3) and interquartile ranges for scores were narrow. The majority of wait times were within 10 to 15 minutes (113,51/212,921, 53.31%).

**Figure 2:**
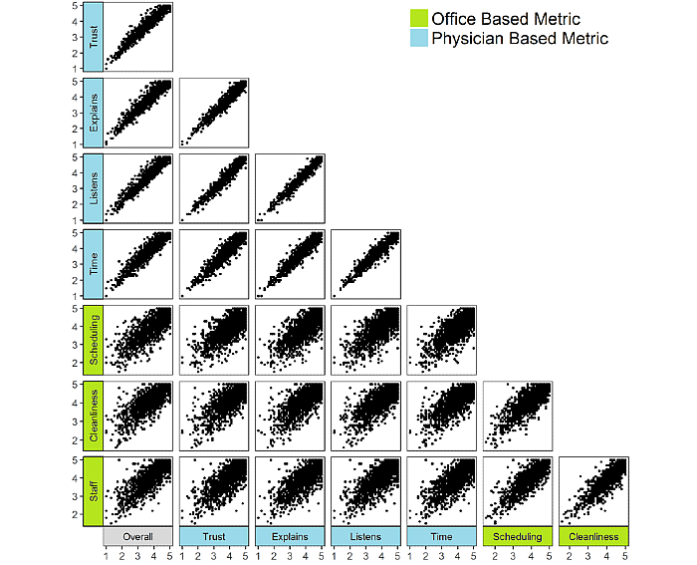
Correlation matrix of physician-based and office-based subdimension ratings.

Physician-based metrics were highly correlated with each other (*r*=.95 to .98, *P*<.001), as were office-based metrics (*r*=.84 to .88, *P*<.001; Figure 2). Correlations between physician-based and office-based ratings were less robust (*r*=.79 to .81, *P*<.001). Overall patient satisfaction correlated more strongly with physician-based metrics (*r*=.95 to .97, *P*<.001) than office-based metrics (*r*=.82 to .84, *P*<.001). Distributions of subdimension scores for providers with overall satisfaction scores of 5.0, 4.0, 3.0, and 2.0 were narrow, with interquartile range of subdimensions spanning a maximum of 0.4 points for physician-based subdimensions and 0.8 points for office-based subdimensions (Table 2).

**Table 1:** Physician demographics (n=212,933).

Characteristics		Values
**Physician specialty group, n (%)**		
	Medical specialties	128,678 (60.43)
	Allied health providers	11,724 (5.51)
	Surgical specialties	72,531 (34.06)
**Geographical region, n (%)**		
	Midwest	44,069 (20.72)
	Northeast	45,616 (21.45)
	South	80,751 (37.96)
	West	42,265 (19.87)
**Year of graduation, n (%)**		
	1945-1954	57 (0.03)
	1955-1964	1579 (0.74)
	1965-1974	1,3475 (6.33)
	1975-1984	47,738 (22.42)
	1985-1994	64,498 (30.29)
	1995-2004	61,338 (28.81)
	2005-2014	20,349 (9.56)
	2015-2016	61 (0.03)
	Unknown	3838 (1.80)
Overall patient satisfaction, median (IQR)		4.10 (3.40, 4.60)
**Physician-based subdomains, median (IQR)**		
	Trust (level of trust in provider’s decision)	4.20 (3.60, 4.60)
	Explains (how well provider explains medical conditions)	4.20 (3.60, 4.60)
	Listens (how well provider listens and answers questions)	4.20 (3.60, 4.60)
	Time (spends appropriate amount of time with patients)	4.20 (3.60, 4.60)
**Office-based subdomains, median (IQR)**		
	Scheduling (ease of scheduling urgent appointments)	4.20 (3.60, 4.60)
	Cleanliness (office environment, cleanliness, comfort)	4.30 (3.90, 4.70)
	Staff (staff friendliness and courteousness)	4.20 (3.70, 4.60)
**Total wait time in minutes, n (%)**		
	<10	31,177 (14.64)
	10-15	113,517 (53.31)
	16-30	54,412 (25.55)
	31-45	12,907 (6.06)
	Over 45	908 (0.43)
	Unknown	12 (0.01)

**Table 2:** Distribution of subdimension scores for providers with overall satisfaction scores of 5.0, 4.0, 3.0, and 2.0.

Subdimension		5.0	4.0	3.0	2.0
**Physician-based, median (IQR)**					
	Trust	5.0 (5.0-5.0)	4.1 (4.0-4.2)	3.2 (3.0-3.3)	2.3 (2.1-2.4)
	Explains	5.0 (5.0-5.0)	4.1 (4.0-4.3)	3.2 (3.0-3.3)	2.2 (2.1-2.4)
	Listens	5.0 (5.0-5.0)	4.1 (4.0-4.2)	3.2 (3.0-3.3)	2.2 (2.0-2.3)
	Time spent	5.0 (5.0-5.0)	4.1 (4.0-4.3)	3.2 (3.0-3.4)	2.3 (2.0-2.4)
**Office-based, median (IQR)**					
	Scheduling	4.8 (4.7-5.0)	4.1 (3.9-4.3)	3.4 (3.1-3.7)	2.7 (2.3-3.1)
	Office cleanliness	4.9 (4.8-5.0)	4.3 (4.1-4.5)	3.7 (3.4-4.0)	3.0 (2.2-2.4)
	Staff friendliness	4.9 (4.8-5.0)	4.1 (3.9-4.4)	3.5 (3.2-3.8)	2.8 (2.4-3.1)

Factor analysis was used to identify latent clusters of variables predicting overall patient satisfaction. One-, 2-, and 3-factor solutions were tested. Although a 1-factor solution explains the majority of the variance, the second factor explains an additional ~10% variance, where the third factor provides negligible information, which supports the results of the parallel analysis (Figure 1, Table 3). In the 2-factor model, two discrete clusters of variables exceeded the a priori defined loading cutoff of ≥0.70 (Table 3). These clusters corresponded directly with physician-based metrics (factor 1 loading values 0.84 to 0.88) and office-based metrics (factor 2 loading values 0.76 to 0.84). Confirmatory factor analysis showed that the individual subdomains loaded successfully upon the a priori hypothesized latent factor structure (Table 4).

**Table 3:** Factor loadings from exploratory factor analysis.

Physician ratings subdimension	1-Factor solution	2-Factor solution		3-Factor solution		
	Factor 1	Factor 1	Factor 2	Factor 1	Factor 2	Factor 3
Level of trust in provider’s decision	0.99	0.86	0.48	0.84	0.52	0.11
How well provider explains medical conditions	0.99	0.87	0.47	0.85	0.51	N/A^a^
How well provider listens and answers questions	0.99	0.88	0.46	0.86	0.50	N/A
Spends appropriate amount of time with patients	0.98	0.84	0.49	0.82	0.53	N/A
Ease of scheduling urgent appointments	0.81	0.50	0.76	0.47	0.78	N/A
Office environment, cleanliness, comfort, etc	0.81	0.49	0.78	0.46	0.80	N/A
Staff friendliness and courteousness	0.82	0.46	0.84	0.43	0.86	N/A
Proportion variance explained	0.84	0.53	0.40	0.49	0.44	0.00
Cumulative variance explained	0.84	0.53	0.93	0.49	0.93	0.93

^a^N/A: Not applicable.

**Table 4:** Factor loadings from confirmatory factor analysis.

Latent factor and items		Loadings	*P* value
**Physician-based metrics**			
	Level of trust in provider’s decision	0.98	<.001
	How well provider explains medical conditions	0.99	<.001
	How well provider listens and answers questions	0.99	<.001
	Spends appropriate amount of time with patients	0.98	<.001
**Office-based metrics**			
	Ease of scheduling urgent appointments	0.92	<.001
	Office environment, cleanliness, comfort, etc.	0.93	<.001
	Staff friendliness and courteousness	0.95	<.001

In univariable linear regression analysis, all physician-based metrics and office-based metrics were associated with overall satisfaction (Table 5). The physician-based subdimensions most strongly associated with overall satisfaction were trust in physician and ability to explain, with overall satisfaction ratings increasing by 1.05 (95% CI 1.04 to 1.05) and 1.03 (95% CI 1.03 to 1.03) points for each point increase in subdimension score, respectively. The office-based subdimension most strongly associated with overall satisfaction was office cleanliness, with overall satisfaction ratings increasing by 1.09 (95% CI 1.09 to 1.1) for each point increase in office cleanliness score. Stepwise increases in office wait times were strongly associated with worsening overall satisfaction ratings; for example, compared with those with total wait time under 10 minutes, a wait time of 31 to 45 minutes was associated with a decrease of –1.35 (95% CI –1.37 to –1.34) in overall satisfaction score. Since subdimension scores were highly correlated, latent factors identifying physician-based metrics and office-based metrics were used in multivariable analysis. In multivariable linear regression, physician-based metrics (0.65; 95% CI 0.65 to 0.65, *P*<.001) were more strongly associated with overall satisfaction than office-based metrics (0.42; 95% CI 0.42 to 0.42, *P*<.001), and the association of office wait times was strikingly diminished (Table 5).

While physician demographics such as practice region and years in practice were also associated with overall satisfaction score in univariable analysis, none were meaningfully associated with overall satisfaction in multivariable analysis (Table 5).

**Table 5:** Univariable and multivariable linear regression model predicting overall satisfaction.

Characteristics		Univariable		Multivariable	
		β (95% CI)	*P* value	β (95% CI)	*P* value
**Physician specialty group, n (%)**					
	Medical specialties	Reference		Reference	
	Allied health providers	0.48 (0.47 to 0.50)	<.001	0.00 (–0.01 to 0.00)	.20
	Surgical specialties	0.18 (0.17 to 0.19)	<.001	0.01 (0.01 to 0.01)	<.001
**Geographical region, n (%)**					
	Midwest	Reference		Reference	
	Northeast	0.04 (0.03 to 0.05)	<.001	0.00 (0.00 to 0.00)	.30
	South	0.00 (–0.01 to 0.01)	.53	–0.01 (–0.01 to 0.00)	<.001
	West	–0.08 (–0.09 to –0.06)	<.001	0.00 (0.00 to 0.00)	.01
**Year of graduation, n (%)**					
	1945-1954	Reference		Reference	
	1955-1964	–0.06 (–0.27 to 0.15)	.60	0.01 (–0.03 to 0.05)	.61
	1965-1974	–0.04 (–0.25 to 0.16)	.68	0.01 (–0.03 to 0.05)	.63
	1975-1984	0.04 (–0.16 to 0.25)	.68	0.01 (–0.04 to 0.05)	.79
	1985-1994	0.08 (–0.13 to 0.28)	.46	0.00 (–0.04 to 0.04)	.92
	1995-2004	0.18 (–0.02 to 0.39)	.08	0.00 (–0.04 to 0.04)	.92
	2005-2014	0.32 (0.11 to 0.53)	.002	0.01 (–0.04 to 0.05)	.77
	2015-2016	0.72 (0.44 to 1.01)	<.001	0.01 (–0.04 to 0.07)	.62
**Physician-based metrics**				0.65 (0.65 to 0.65)^a^	<.001
	Trust	1.05 (1.04 to 1.05)	<.001	N/A^b^	N/A
	Explains	1.03 (1.03 to 1.03)	<.001	N/A	N/A
	Listens	1.01 (1.01 to 1.01)	<.001	N/A	N/A
	Time	1.02 (1.02 to 1.02)	<.001	N/A	N/A
**Office-based metrics**				0.42 (0.42 to 0.42)^a^	<.001
	Scheduling	0.99 (0.99 to 0.99)	<.001	N/A	N/A
	Cleanliness	1.09 (1.09 to 1.10)	<.001	N/A	N/A
	Staff	1.02 (1.02 to 1.02)	<.001	N/A	N/A
**Total wait time in minutes**					
	<10	Reference		Reference	
	10-15	–0.43 (–0.44 to –0.42)	<.001	–0.01 (–0.01 to –0.01)	<.001
	16-30	–0.89 (–0.89 to –0.88)	<.001	–0.02 (–0.03 to –0.02)	<.001
	31-45	–1.35 (–1.37 to –1.34)	<.001	–0.06 (–0.06 to –0.05)	<.001
	>45	–1.90 (–1.95 to –1.85)	<.001	–0.10 (–0.11 to –0.09)	<.001

^a^Factor score from exploratory factor analysis.

^b^N/A: Not applicable.

## Discussion

### Principal Findings

Online physician ratings have been steadily gaining popularity, with physicians rated a median of 7 times across commercially available websites [1,28]. More consumers are aware of these ratings and are using them as the primary source of information to guide their health care decisions [7,29]. In addition, more physicians are now being rated across multiple platforms [2,3,30], yet despite the groundswell of interest and uptake of online ratings, very little data exist to support their validity and utility in assisting consumers choose better physicians [4,13,31-33]. In this analysis of a large sample of quantitative online reviews, we found that physician-based subdimensions were very highly correlated with one another, demonstrating that consumers rarely differentiate between the commonly rated subdimensions of physician care. Office-based subdimensions of care were also found to be highly correlated with one another. However, there was more heterogeneity observed when comparing physician-based subdimensions with office-based subdimensions, suggesting that patients are better at parsing between the perceived quality of the physician versus their office staff. Factor analysis objectively supports this contention, clearly identifying two discrete factors predicting overall satisfaction, one clustered around physician-based care and one around office-based aspects of care. In multivariable regression analysis, the composite factors measuring physician- and office-based care far eclipsed other demographics in prediction of overall satisfaction in terms of magnitude. We believe this data suggests that physician ratings should be simplified to two simple metrics: one evaluating physician-based care and one evaluating office-based services.

The principal finding of our study is that commonly rated subdimensions of physician-based care are highly correlated with one another, which calls into question their utility over a single measure of satisfaction with the physician. Either the vast majority of physicians are consistently all good, average, or bad across all categories of care or consumers are unable to discriminate between the measured characteristics of their physicians. Since the former explanation does not seem likely, we favor the latter explanation. Kadry et al [34] also found a high correlation between various subdimensions of care across multiple rating websites in their analysis of 4999 total ratings and argued for a single rating for physician-based care. Indeed, based on our more comprehensive data analysis, a single measure of satisfaction with the physician and a single measure satisfaction with the office staff would suffice. Reducing the number of ratings could improve the understandability of these reviews and increase response rates [35,36].

An additional explanation for why the physician-based subdimension scores in our study may be highly correlated is the fact that they are measuring a similar construct: bedside manner. While the office-based subdimensions measure discrete, quantifiable characteristics such as office environment and ease of scheduling appointments, the physician-based subdimensions measure aspects of relationship building between doctor and patient [37,38]. It is difficult to conceive of a physician who would be superb at one aspect of relationship building (eg, listening and answering questions) and abysmal at another (eg, building trust). To our knowledge, none of the subcomponent scores of online ratings have undergone rigorous psychometric validation to determine if they are measuring distinct constructs. Although our study is not a psychometric assessment, it does suggest deficiency in discrimination between the subdimension scores by their overwhelming correlation with each other. As an extension of this line of thought, an alternative to reducing physician ratings to single measures of physician- and office-based care would be to identify components of care that patients can differentiate between using rigorous psychometric techniques.

While online ratings may be flawed, they are clearly an important source of direct consumer feedback, and we believe that these ratings have the potential to give physicians important quality improvement feedback [4,6,14,39,40]. While composite measures of physician- and office-based care were the predominant predictors of overall satisfaction in multivariable linear regression, there were some other notable characteristics that are worth mentioning. In univariate analysis, incremental increases in wait time predicted significantly worse ratings. On just a 5-point Likert scale, physicians with wait times over 45 minutes had an average of a 1.9-point lower rating compared with physicians with wait times under 10 minutes. Even physicians with wait times of just 10 to 15 minutes had nearly a half point decrease in ratings. As physician ratings do not fall under a normal distribution, these decreases can have a significant impact in the online perception of a physician when compared with his or her peers [18]. Interestingly, physician age and experience did not seem to affect their ratings with the exception of physicians who had graduated between 2005 and 2016. While surveys have shown that patients generally prefer physicians toward the middle of their career, this younger group actually had higher ratings despite less clinical experience. Gao et al [2] also had similar findings from another physician review website [41]. Younger physicians may have a better understanding of their online presence and focus more time identifying ways to improve their rating. Nonetheless, as mentioned above, physician-based metrics and office-based metrics far outstripped these demographic predictors of overall satisfaction in our multivariable model.

### Limitations

There are several limitations to our study. We aggregated data only from one website. However, this is the most frequented physician rating website with a large, heterogeneous mixture of physicians from across the United States [34]. Aggregation of data from multiple websites may be impractical given the large number of websites and various rating methods. In addition, these reviews naturally have an implicit selection bias and may not always be authentic. To minimize this bias, we only included physicians with more than the median number of reviews (4).

### Conclusions

In this analysis of the ratings of 212,933 providers, we found that consumers do not often differentiate between commonly assessed physician-based subdimensions of care. Physicians were most often scored in a monochrome fashion: scores all good, average, or bad. Office-based subdimensions of care were also highly correlated and were scored in a similarly monochrome fashion. In multivariable analysis, composite latent factors identifying physician-based metrics and office-based metrics were both independently associated with overall satisfaction scores, eclipsing all other physician demographic predictors in terms of magnitude. Based on this, we question the utility of commonly used subdimension scores and instead recommend a single measure of satisfaction for the physician and a single measure of satisfaction for the office staff. Alternatively, further research should be conducted to identify qualities of physicians and office staff that consumers are well positioned to evaluate and are meaningful to patient experience.

## Appendix

Multimedia Appendix 1 Healthgrades.com homepage and sample physician review.
