# Supplementary material for: Correlation of Online Physician Rating Subscores and Association With Overall Satisfaction: Observational Study of 212,933 Providers
Source: J Med Internet Res. 2020 Oct 27;22(10):e11258. doi: 10.2196/11258 (PMC7655464; doi:10.2196/11258)

Healthgrades Home Page ([www.healthgrades.com](http://www.healthgrades.com)).


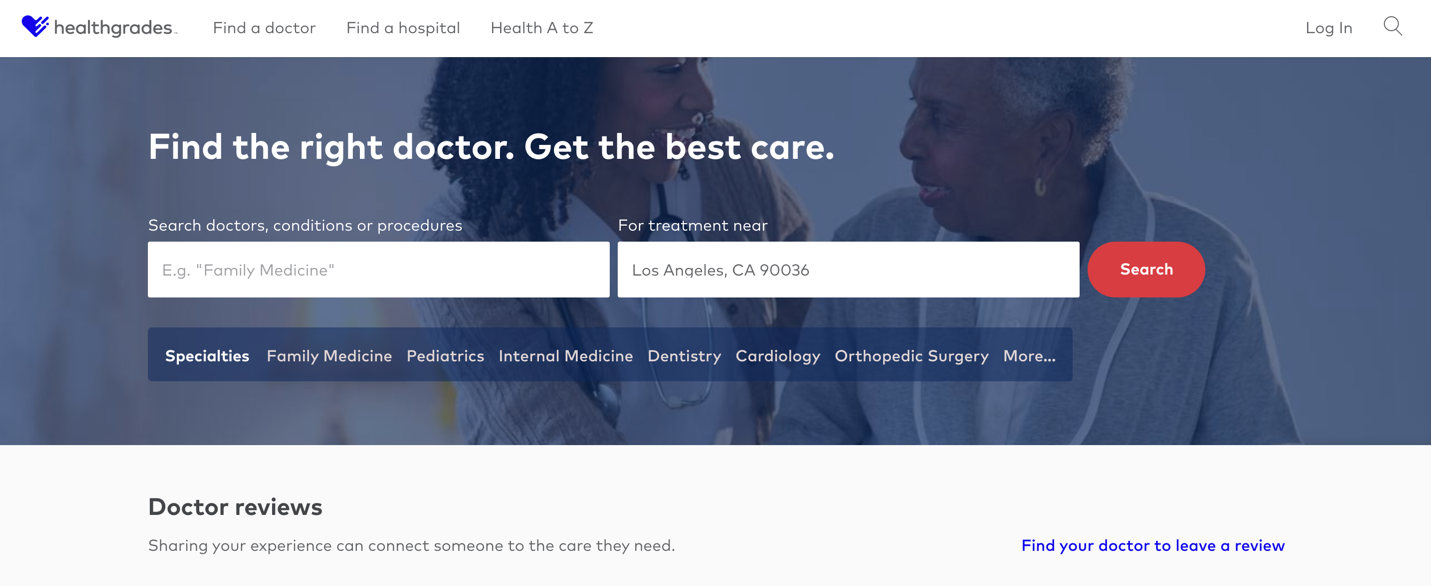


Sample Physician Review.


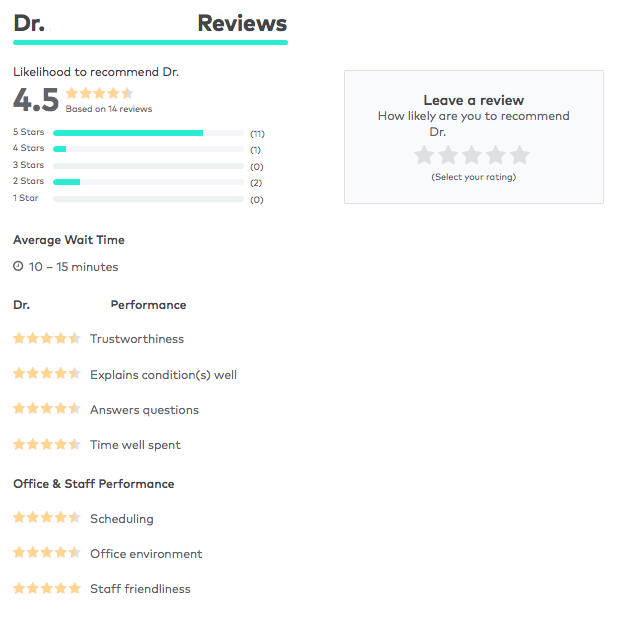

Supplement: Multimedia Appendix 1 [file jmir_v22i10e11258_app1.docx]
